# An implantable helmet for studying repeat TBI

**DOI:** 10.1016/j.mex.2020.101142

**Published:** 2020-11-14

**Authors:** Katelynn Ondek, Steven Lucero, Marike Zwienenberg, Gene Gurkoff

**Affiliations:** a Department of Neurological Surgery, University of California, 1515 Newton Ct, Davis, CA 95618, United States; b Department of Biomedical Engineering, University of California, Davis, CA 95618, United States

**Keywords:** Repeat injury, Sports injury, mTBI

## Abstract

An estimated 3.8 million traumatic brain injuries (TBI) occur each year, the majority classified as mild. Interest in models of mild and repeat mild TBI has grown due to reports of lasting morbidity following sports- or combat-related injury. There remains a paucity of data linking cellular or systems-related mechanisms to behavioral outcomes following repeat mild TBI, particularly in adolescent and adult rats. It is critical, therefore, to develop flexible models to evaluate which parameters of injury are associated with brain vulnerability or poor chronic outcome compared to normal recovery. While there are several existing models of repeat mild TBI in rodents, studying the effects of multiple hits has been complicated by the need for multiple survival surgeries, extensive pre-injury anesthesia time, and limitations due to animal skull thickness.

• We developed a chronic “helmet” implant by combining aspects of the Impact Acceleration and Controlled Cortical Impact models.
• Implants were performed days before injury, allowing us to decouple surgery from TBI. Critically, by pre-implanting the animals, only minimal anesthesia was required to position them under the impactor.
• The implant allows for flexibility in the number and severity of injuries and interval between impacts.

Specifications table

**Table:** 

Subject Area	Neuroscience
More specific subject area	Rodent models of traumatic brain injury
Method name	Implant for repeat traumatic brain injury
Name and reference of original method	Marmarou, A., Foda, M.A., van den Brink, W., Campbell, J., Kita, H., and Demetriadou, K. (1994). A new model of diffuse brain injury in rats. Part I: Pathophysiology and biomechanics. J. Neurosurg. *80*, 291–300. Dixon, C.E., Clifton, G.L., Lighthall, J.W., Yaghmai, A.A., and Hayes, R.L. (1991). A controlled cortical impact model of traumatic brain injury in the rat. J. Neurosci. Methods *39*, 253–262. Huh, J.W., Franklin, M.A., Widing, A.G., and Raghupathi, R. (2006). Regionally distinct patterns of calpain activation and traumatic axonal injury following contusive brain injury in immature rats. Dev. Neurosci. *28*, 466–476. Prins, M.L., Hales, A., Reger, M., Giza, C.C., and Hovda, D.A. (2010). Repeat traumatic brain injury in the juvenile rat is associated with increased axonal injury and cognitive impairments. Dev. Neurosci. *32*, 510–518.
Resource availability	Autodesk Inventor file will be shared if contacted.

## Method details

### Animals and groups

Juvenile (P21) male Sprague Dawley outbred rats were purchased from Envigo (Livermore, CA). Rats were given 10 days to habituate to the vivarium, during which they were handled daily. Initial pilot studies (*n* = 12) were conducted prior to launching our first study. These pilots consisted of two versions of the implant, each with a unique configuration of skull screws to determine how to create a rigid and stable helmet that could survive multiple injuries without repair (see Supplemental Information). Ultimately*,* we designed a study in which animals were run in cohorts of 6–10, with each implant lasting four weeks. Within each cohort, animals were randomly assigned to Single Injury (*n* = 12), Repeat Injury (*n* = 14), or Sham Injury control (*n* = 12) groups. One animal from the repeat injury group was sacrificed due to complications following the third injury (final Repeat Injury *n* = 13). All procedures adhere to the National Institutes of Health guidelines and were approved by the University of California, Davis Institutional Animal Care and Use Committee.

### Implant design

We combined aspects of the Impact Acceleration (IA) and Controlled Cortical Impact (CCI) models of traumatic brain injury (TBI) to develop a new model for studying repeated mild TBI (mTBI) in adolescent rats. Multiple iterations of the implant were tested prior to arriving at the final design (see Supplementary Materials and Additional Information). We designed a 3D-printed cap that could be combined with the stainless-steel disc created for the IA model to create a “helmet” that could be surgically implanted on the rodent's head. *Once implanted, this helmet allowed for repeat injuries using the CCI device without multiple survival surgeries and minimizing the required pre-injury anesthesia time.*

**Fig. 1:**
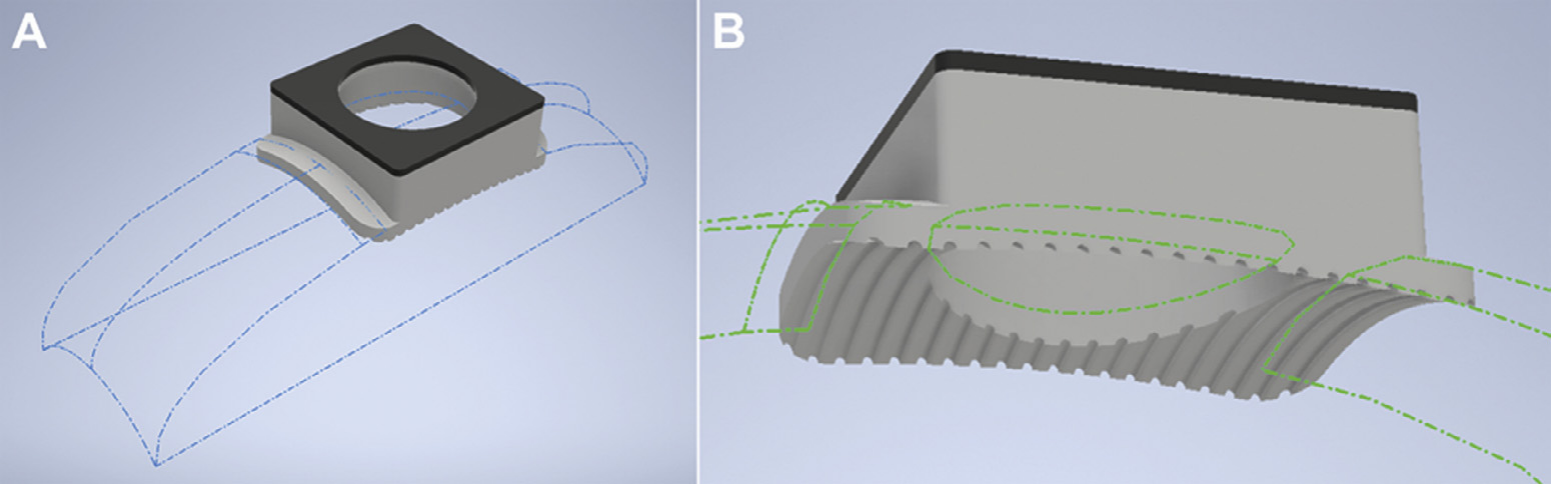
CAD Renderings of the Phantom Skull. A P31 skull was used to create a phantom in Autodesk Inventor Pro that could be used to shape the ventral surface of the implant (A). Viewed from the ventral plane, the base on the cap conforms well to the shape of the adolescent skull (B).

**Fig. 2:**
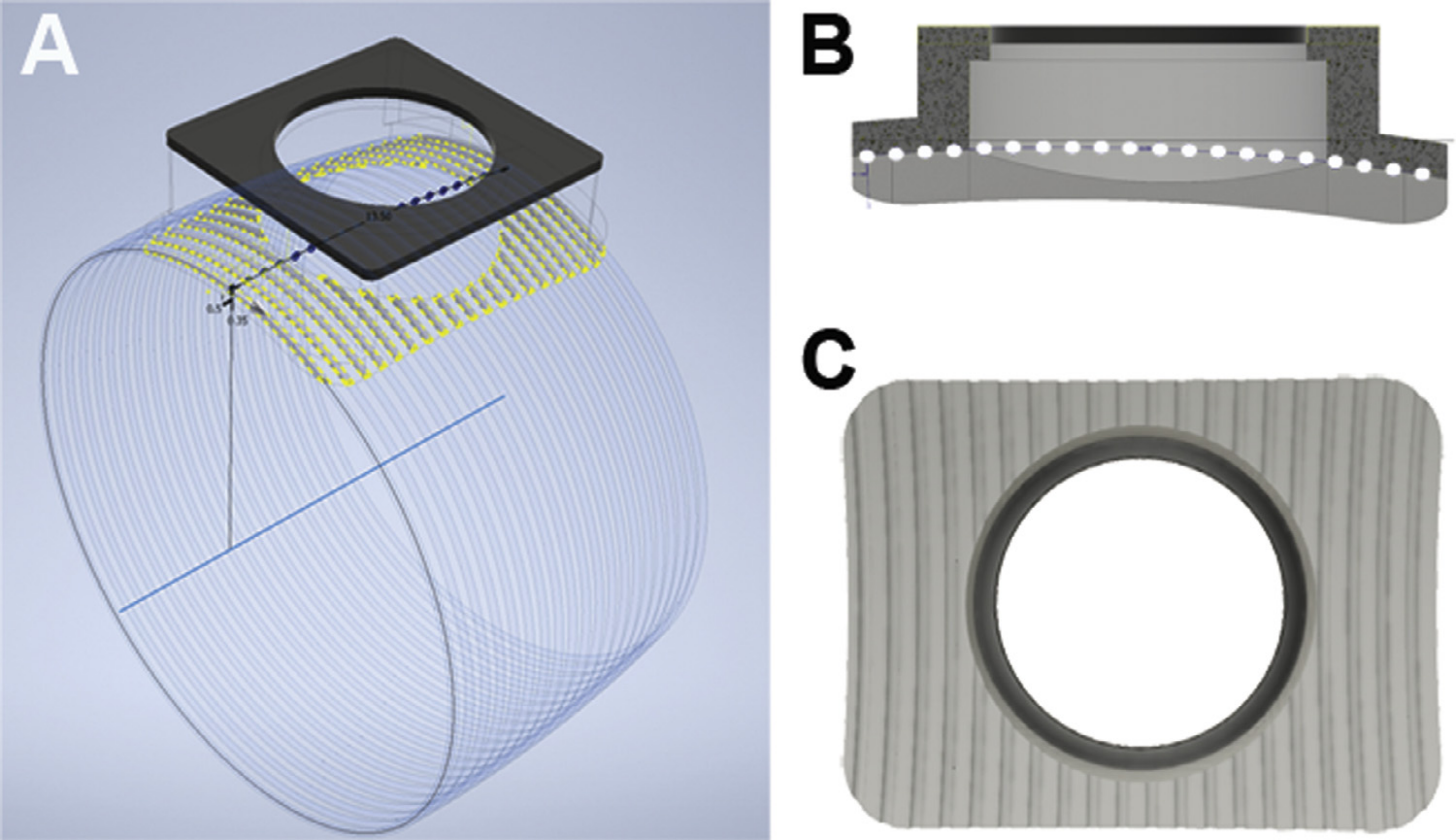
CAD Renderings of the Groove Design. To accommodate the curvature of the implant, a phantom circle was created, consisting of 0.35 mm diameter circles (A) spaced 0.51 mm apart on the perimeter (yellow dots). Ultimately half circles were cut into the plastic (B) resulting in regular grooves along the ventral surface of the implant (C).

In order for the cap to sit stably, and to allow maximal surface area for bonding, we had to consider the curvature of the adolescent rat skull. A P31 male Sprague Dawley rat was euthanized with Euthasol (100 mg/kg) followed by rapid decapitation. After the soft tissue was carefully removed from the skull, the bones were soaked in cold water with dish detergent and then in solution of 1 part 30% H_2_O_2_: 1 part H_2_O to thoroughly disinfect them. Measurements taken from this skull were used to design a phantom that could then be imported into Autodesk Inventor Pro (Autodesk Inc., San Rafael, CA). The skull allowed us to incorporate the curvature and size specific to the developmental time point we proposed to test in our initial studies (Fig. 1). To further maximize surface area for bonding the skull to the plastic, 0.175 mm radius half-circles with 0.51 mm spacing were cut along the entire ventral face of the implant. (Fig. 2).

**Fig. 3:**
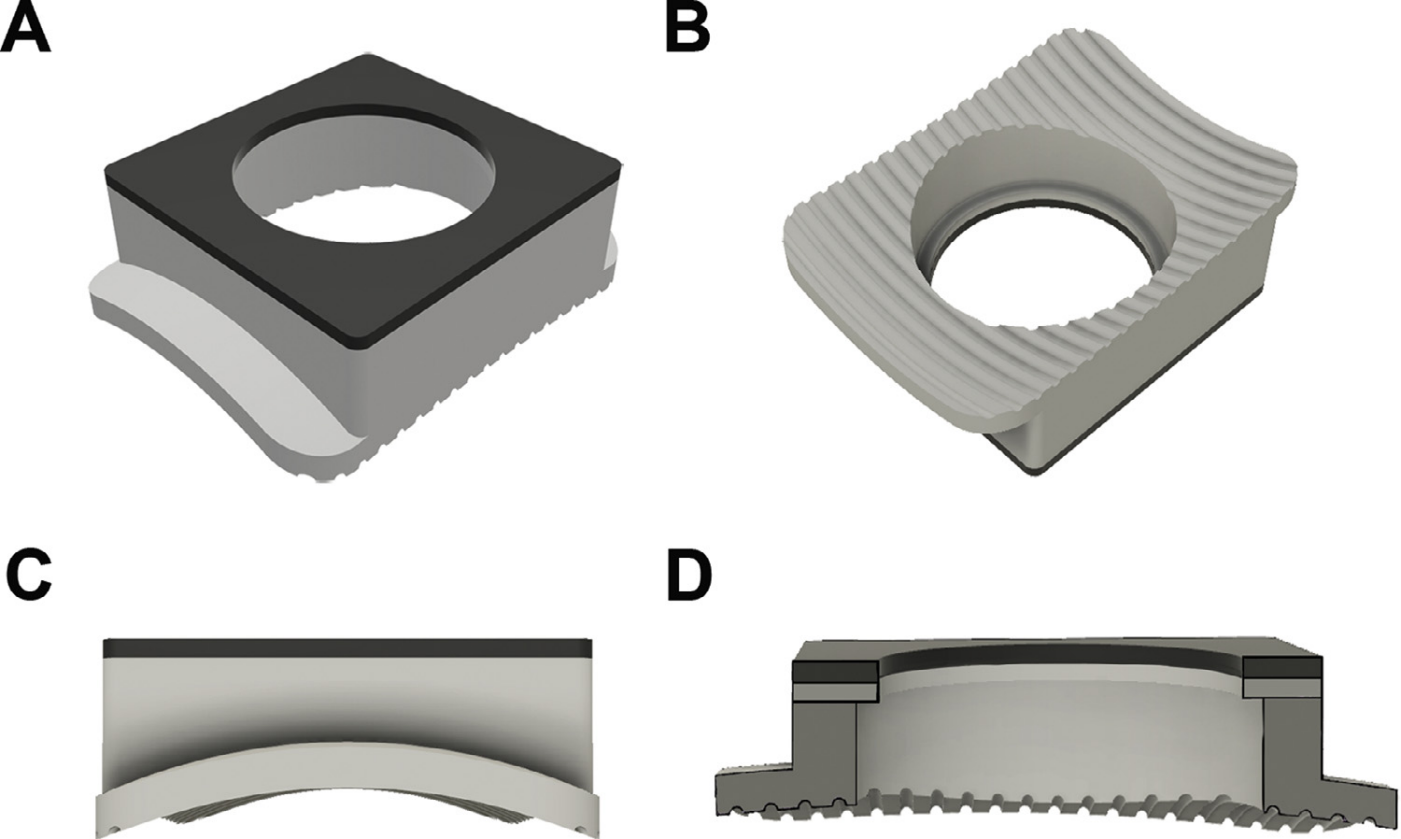
CAD renderings of the plastic implant. From the dorsal plane, the TangoBlackPlus substrate sits atop the implant allowing for tracking. The dorsal surface has a 9.33 mm cutout. Wings extend on the ventral surface (A). From the ventral plane, the wider 10.5 mm cutout is apparent, as well as the half-circle grooves included to increase surface area (B). When looking from the anterior aspect, the curve of the implant is appreciable (C). A medial cross-section highlights the layers of VeroWhitePlus and TangoBlackPlus, as well as the different diameter of the apertures on the dorsal and ventral aspects for the implanted metal disk (D).

The implant was fabricated on an Objet Connex 260v (Stratasys, Eden Prairie, MN) polyjet printer from VeroWhitePlus, and a 0.5 mm layer of TangoBlackPlus (Stratasys) was adhered to the top of the cap for automated video tracking purposes (Fig. 3A). The main body of the implant was 13.0 mm long by 13.0 mm wide by 4.610 mm high (see Supplementary Table 1 for full dimensions). To increase surface area, wings extended from the ventral aspect of the anterior and posterior faces, measuring 2.005 mm long by 13.0 mm wide by 1.052 mm high (Fig. 3A&C). There was a 10.5 mm cutout on the ventral side to accommodate a stainless steel disc (Fig. 3B; diameter = 10 mm, height = 3 mm; Custom Design and Fabrication South, Petersburg VA). The dorsal face had included an aperture through which the impactor could directly strike the disc, as well as a rim around the disc to secure it in place and prevent it from dislodging upon impact. The diameter of the opening on the dorsal face was set at 9.3 mm, creating a 0.6 mm wide by 0.5 mm thick rim of plastic on all sides of the stainless-steel disc (Fig. 3D).

### Implant surgery

All caps were cold-sterilized for a minimum of 12 h in Cetylcide-G (33 mL) + Cetylcide-G Diluent (Cetylite, Pennsauken, NJ) diluted in sterile, deionized water (946 mL) and then rinsed 3 × 1 min in sterile, deionized water prior to implantation. Caps were discarded after a single use.

**Fig. 4:**
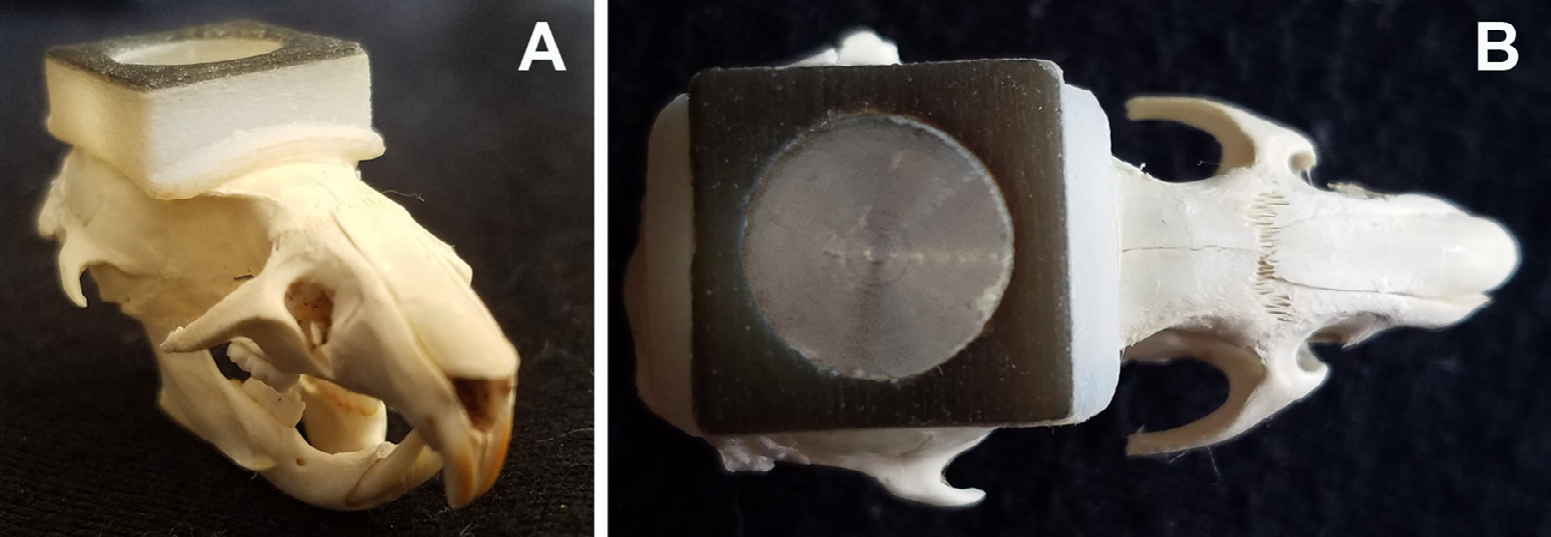
The implant was centered along midline, with the curvature wrapping around the dorsal aspect of the rat skull (A). From the top, the stainless-steel disk would is centered midway between lambda and bregma (B). The wings stretching anterior and posterior provide extra surface both for binding to the skull with super glue (ventral) and embedding in acrylic (dorsal).

Animals were pretreated with carprofen (5 mg/kg, s.c.; Dechra Pharmaceuticals, Northwitch, United Kingdom) 30 min prior to initiation of anesthesia. As previously described [1], rats were anesthetized in 4% isoflurane (in air). After reaching a surgical plane of anesthesia, the animals’ heads were shaved, and they were placed on a heating pad (37.0 ± 1.5°) with anesthesia maintained (1.5 - 3% isoflurane in 2 NO_2_:1 O_2_) via a nose cone. The skin was then sterilized using alternating alcohol and betadine wipes (3X each), followed by injection of a local anesthetic (0.1 mL of 0.25% bupivacaine s.c.) directly over the incision. A midline scalp incision was made, and the skin was retracted to expose the dorsal cranial surface. To increase the surface area of the skull, we applied pharmaceutical grade dental etchant (35% phosphoric acid) for 60 s and then rinsed with sterile saline solution. The skull surface was dried first with sterile gauze and then by air. In order to create an anchor for the implant, a #0–80 stainless steel screw was embedded in the skull over the cerebellum. We added several drops of super glue gel to the ridged side of the metal disc and then affixed it to the skull, centering it halfway between bregma and lambda. After the disc was in place, we applied super glue gel to the bottom of the custom-designed implant, and placed it directly over the disc (Fig. 4). In order to form a very stable implant, we applied a thin layer of C&B-Metabond Adhesive Luting Cement (Parkell, Inc.) followed by a thick layer of the acrylic. No sutures were required as the acrylic formed a tight seal with the skin that lasted the duration of the experiment. In order to minimize pain and inflammation, the nonsteroidal anti-inflammatory (NSAID) carprofen (s.c.) was administered at 24 and 48 hrs post-implant, and the local anesthetic bupivacaine was applied around the implant at 2–5 hrs and 1-day post-surgery.

### Inducing traumatic brain injury

On P35, all rats – including Shams – were anesthetized in a chamber for exactly 3 min using 4% isoflurane in air, followed by exactly 2 min of 2% isoflurane (in 2 NO_2_:1 O_2_) via nose cone. During the last two minutes of anesthetization, rats were positioned on a stiff Styrofoam pad directly under the CCI device (eCCI-6.3, Custom Design and Fabrication South, Petersburg, VA), and the 5 mm stainless steel impactor tip was lowered and zeroed with the metal disc. In order to reduce the influence of anesthesia on injury, we terminated the use of isoflurane exactly 30 s prior to injury. Based on pilot data, we programmed the impactor to deliver a 5 m/s impact, with the tip moving 5 mm depth past the zero point. In order to validate the reproducibility, we recorded the measured velocity and depth provided by the instrument. On the first day of injury only animals in the Repeat injury group received an impact, whereas animals in the Sham and Single injury groups received similar durations of anesthesia and were positioned similarly under the CCI device, but the CCI device was not triggered. This procedure was repeated on P38, again with only the Repeat injury group receiving an impact. Both the Repeat and the Single injury group received a mechanical insult on P41. Again, *all* animals received similar durations of anesthesia on P35, P38, and P41 and were placed on a heating pad on the Styrofoam pad regardless of whether they received an injury.

## Method validation

### Consistency of impact

To ensure the model had limited variability between injuries, we compared the velocities and depths of each impact, as measured by the CCI controller. Depth and velocity of the impactor were programmed to 5 mm and 5 m/s, respectively, but actual values measured by the controller were variable. As each impact was unique, we considered each of the injuries as an individual measure and compared values between days 35 (Repeat group only), 38 (Repeat group only) and 41 (both Single and Repeat groups). There were no significant differences in impact velocity (F_(3,48)_ = 0.7695, *p* = 0.5168) or depth (F_(3,48)_ = 0.5169, *p* = 0.6727) across injuries based on a one-way ANOVA [1].

### Biological response to injury

To ensure that the implant did not completely absorb the force from impact, we analyzed acute biological response following injury (Table 1). Immediately following impact, or 30 s after termination of isoflurane for non-injury groups, all animals were evaluated for toe pinch (every 5 s) and righting (continuous) reflexes. Analyzing sham, single injury (a combination of the Repeat Injury group on P35 and the Single Injury group of P41), and three injuries (the Repeat Injury group on P41) with an one-way ANOVA, we identified a significant main effect of injury number (F_(2,93)_ = 8.3, *p* < 0.001). A Bonferroni post-hoc analysis found a significant increase in toe pinch response in rats following their third injury as compared to both sham (*p* < 0.001) and first injury (*p* < 0.05). There were no effects of single or repeat injury on righting reflex (*p* = 0.93).

**Table 1:** Immediate biological responses to injury.

	Sham Injury	First Injury	Third Injury
Toe Pinch Reflex (s)	5.526 ± 0.205	6.042 ± 0.519	8.333 ± 1.281
Righting Reflex (s)	58.321 ± 3.367	59.957 ± 5.508	60.273 ± 8.599

Immediately following injury, or 30 s after sham injury, we measured latency to the return of toe pinch reflex in 5 s intervals, as well as the total time (s) for rats to return to sternal recumbency. All results presented as arithmetic mean ± SEM.

Within the Single Injury group, there was a significant difference in weight gain between sham injury on P35 (anesthesia only) and injury on P41 (*p* < 0.001), with rats gaining significantly more weight if given anesthesia only (Table 2). However, animals in the Repeat Injury group demonstrated no significant difference in weight gain between their first injury (P35) and third injury (P41) in the repeat injury group. These data suggest that, while an injury temporarily slowed weight gain, there was no cumulative effect of three injuries.

**Table 2:** Weight change following injury.

	P36	P42
Single Injury Group	5.762 ± 0.332	3.081 ± 0.346***
Repeat Injury Group	3.281 ± 0.443	3.233 ± 0.519

We recorded weight change on the first day following an impact or sham injury as a percentage change from weight on the day of injury. Separate paired t-tests were used to compare weight change (%) on P36 and P42 for both the Single and Repeat Injury groups. Data are presented as arithmetic mean ± SEM, ***p<0.001.

### Future considerations of the model

Anesthesia time was set for 5 min for this study, including 3 min at 4% isoflurane in an induction chamber and 2 min at 2% isoflurane to position the animal and set the piston. We believe that it is possible to reduce time in the induction chamber to 2 min and the time needed to position the rat and set the CCI device to 1 min reducing the total anesthesia time to only 3 min.

One of our primary concerns with the current model is related to injury severity. Specifically, the CCI device in the lab has maximal settings of 6 m/s. The combination of the metal disc and the surrounding dental acrylic absorb a considerable amount of energy, protecting the skull and underlying brain much like a helmet would. To increase injury severity or adapt this model to an older rat with thicker skull, we would likely need to increase the amount of force that could be delivered by the impactor.

One other critical variable related to injury severity is the substrate used to support the head during injury. In the initial IA injury, rats rest on a flexible foam, allowing for considerable head rotation in the axial plane and leading to axonal injury [2]. In the UCLA model of closed skull injury (see additional information for more details), the rats are free to move laterally after impact, allowing for some rotational strain, also resulting in axonal damage [3]. For the current project, a rigid Styrofoam was selected, similar to the model generated by Huh and Raghupathi (see additional information). This configuration allows for minimal rotation in the axial plane, and axonal damage was not assessed. Future studies should consider using various substrates ranging from more rigid to more flexible, allowing for degrees of axial or rotational strain and, ultimately, a spectrum of axonal injury and cognitive dysfunction.

Another concern when developing the model was animal health. Ultimately animals did not lose weight following injury. Moreover, animals were sternal in fewer than 10 min following injury and the injured and sham rats were indistinguishable within 15 min. Preliminary rotorod data (data not shown) did not detect any motor deficits following injury. Therefore, future studies should consider shorter intervals (i.e. 1 or 24 hr) between injuries to determine if there are periods of vulnerability following a single impact. Moreover, as we saw a significant increase in toe pinch following a third injury, another possibility is to increase the number of injuries. For example, rats could be exposed to 5 injuries over the course of two to three weeks. Lastly, in our experiment we may have missed an acute behavioral deficit as water maze as not evaluated until one week following injury. As these animals do largerly recover, seemingly within hours, it should be possible to start assessing learning evan as early as 24 hr after an injury.

Finally, this was a pilot study conducted using funds from a small grant. With limited funding, the model was only tested in male rats between the ages of P35-P41. Future studies will need to compare the effects of injury and repeat injury between male and female rats, as well as perhaps in juvenile (P19) or young adult (P55) rats.

## Appendix Supplementary materials

Image, image 1

Image, application 2

## References

[1] Ondek K., Brevnova O., Jimenez-Ornelas C., Vergara A., Zwienenberg M., Gurkoff G. (2020). A new model of repeat mTBI in adolescent rats. Exp. Neurol..

[2] Heath D.L., Vink R. (1995). Impact acceleration-induced severe diffuse axonal injury in rats: characterization of phosphate metabolism and neurologic outcome. J. Neurotrauma.

[3] Prins M.L., Hales A., Reger M., Giza C.C., Hovda D.A. (2010). Repeat traumatic brain injury in the juvenile rat is associated with increased axonal injury and cognitive impairments. Dev. Neurosci..

