# Supplementary figures and images for: An implantable helmet for studying repeat TBI
Source: MethodsX. 2020 Nov 14;7:101142. doi: 10.1016/j.mex.2020.101142 (PMC7726661; doi:10.1016/j.mex.2020.101142)

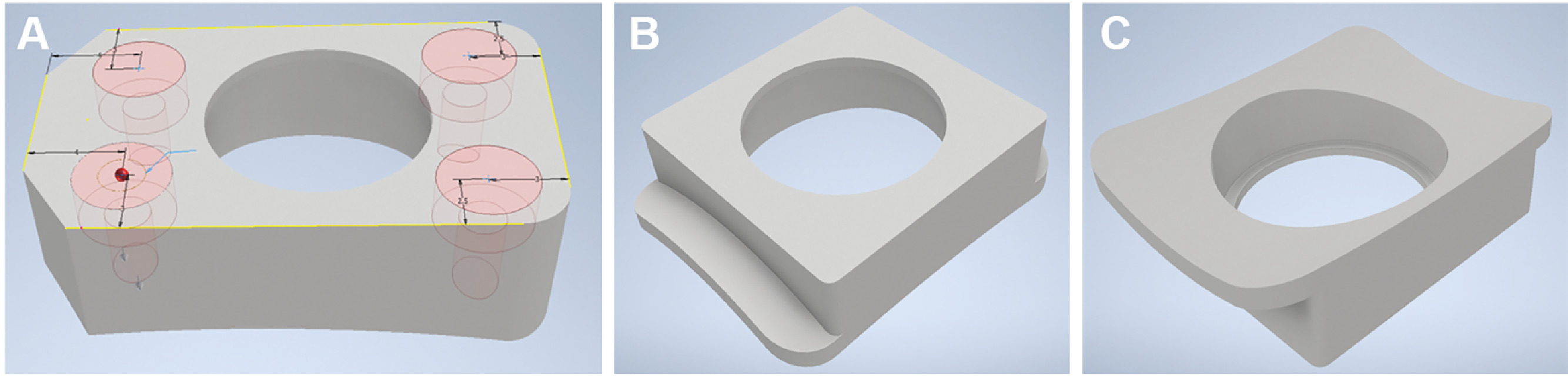

Supplement: Supplementary file 1 [file mmc1.jpg]
