# Supplementary material for: An implantable helmet for studying repeat TBI
Source: MethodsX. 2020 Nov 14;7:101142. doi: 10.1016/j.mex.2020.101142 (PMC7726661; doi:10.1016/j.mex.2020.101142)
Supplement: Supplementary file 2 [file mmc2.docx]

**Supplemental Materials and Additional Information**

*Implant Development*

Initial models of repeat mild TBI in mice (Yoshiyama et al., 2005), and closed skull contusive injury (Huh et al., 2006; Huh and Raghupathi, 2007) and repeat closed skull injury (Huh and Raghupathi, 2007) were first published from the group at University of Pennsylvania. In the model extensively characterized by Drs. Huh and Raghupathi, a rounded silicon tip was attached to the electrically driven piston of the CCI device, replacing the traditional flat bottom, stainless-steel impactor. The modified tip allowed for repetitive midline impact directly to the pediatric rodent skull without risk of skull fracture. A major strength of this method was that a repeat injury could be administered in the absence of a craniotomy. Also, as the head was not fixed in this model, there was some axial rotation associated with the impact, pushing the animals head into a firm Styrofoam pad. However, one of the weaknesses of the model, from an ecological validity standpoint, was that, while injury never happens directly onto the dura, it also typically does not happen directly on the skull either. Instead, the force of impact has to penetrate through both the skin and the skull. Subsequently, a series of publications came out of the University of California, Los Angeles using a CCI device with a standard 5 mm diameter tip and a pneumatic piston. The investigators placed a mask over the skin to allow for precise targeting of the device, the impact was delivered from a 23° angle, and the head was free to move in the direction of the impact, allowing for some rotation. Using an 8 mm penetration of the impactor at 36 psi, possible on the pneumatic but not the electric piston models, the authors demonstrated that repeat injury resulted in axonal injury (Prins et al., 2010), pituitary damage (Greco et al., 2013), and cognitive dysfunction (Prins and Giza, 2012) when administered in a P35 rat. Importantly, they also identified that there was a window of vulnerability following the initial impact where animals had a worse outcome in the second impact was delivered 24, but not 120, hr following the initial insult.

Building off of these initial ideas related to closed skull and repeat injury models, we identified an opportunity for further model development. With growing interest in sports-related concussion and TBI in the military, we decided to generate a model that incorporated a helmet. The impact acceleration model, one of the first rodent models of TBI, took advantage of a stainless-steel disc that was glued directly to the rat’s skull. This disc functions similarly to a helmet, diffusing the force of impact around the skull and preventing skull fracture. The rat was positioned on a foam pad under a long tube, and a weight was dropped down the tube onto the helmet with the rodent pulled away after injury to prevent a second impact. This model was validated to generate a range of injury severities in both juvenile as well as adult rats (Marmarou et al., 1994). One of the more interesting aspects of this injury was that injury could be created not only in juvenile and adolescent rats, but also in older animals with thicker skulls. To date, the closed-skull repeat models are all in pediatric or adolescent rats.

For our initial attempts to develop a closed-skull injury with a helmet, we based our design on the original Huh and Raghupathi papers. Using a cut-out Styrofoam pad to stabilize the head, neck, and body, we performed a midline incision. The metal disc developed for the impact acceleration model was superglued directly to the skull, midline, and halfway between the skull sutures lamda and bregma. The animal was positioned under the piston, and an impact was delivered. With low velocity impacts (2-3 m/s), the cap stayed on the head. With higher impact velocities (5-6 m/s), the cap typically came off the skull on impact. Using this model, we were able to generate a range of injuries in adolescent rats, including those that resulted in no spatial learning deficits as well as those that led to significant deficits in Morris water maze performance (unpublished data). However, several things about this model were suboptimal. Some key examples include 1) loss of the cap with increasing force meant loss of energy to the environment; 2) the duration of anesthesia was roughly 20 min per surgery (n = 31, average of 20.7±0.6 min); 3) repeat injury would require multiple survival surgeries; 4) as with all surgical models of TBI, there was concern about the interaction of non-steroidal anti-inflammatory medications with post-traumatic inflammation.

To solve each of these issues, we developed a 3D printed plastic cap that could permanently house the same metal disc used in the IA mode and be permanently implanted atop the skull. This model would address each of our identified issues. Implanting the helmet would secure the disc so that it would remain affixed to the skull regardless of injury severity. More importantly, the implantation of the helmet could be done days before the injury, allowing us to give anti-inflammatory medication following surgery and to wean animals from the drug at least 24 hours before injury. In addition, implanting the helmet minimized the pre-injury anesthesia time to that required to position the rat under the impactor, reducing the interaction of isoflurane and injury – specifically we went from an average of 20.7±0.6 min to 5 min exactly. Finally, because the helmet was permanently implanted, *only the one survival surgery was needed,* even with repeat injuries.

There were several key variables that were considered and modified over the course of developing the model including: 1) size of the implant, 2) material used to make the implant, and 3) how to secure the implant to the skull to minimize the risk of spontaneous explantation.


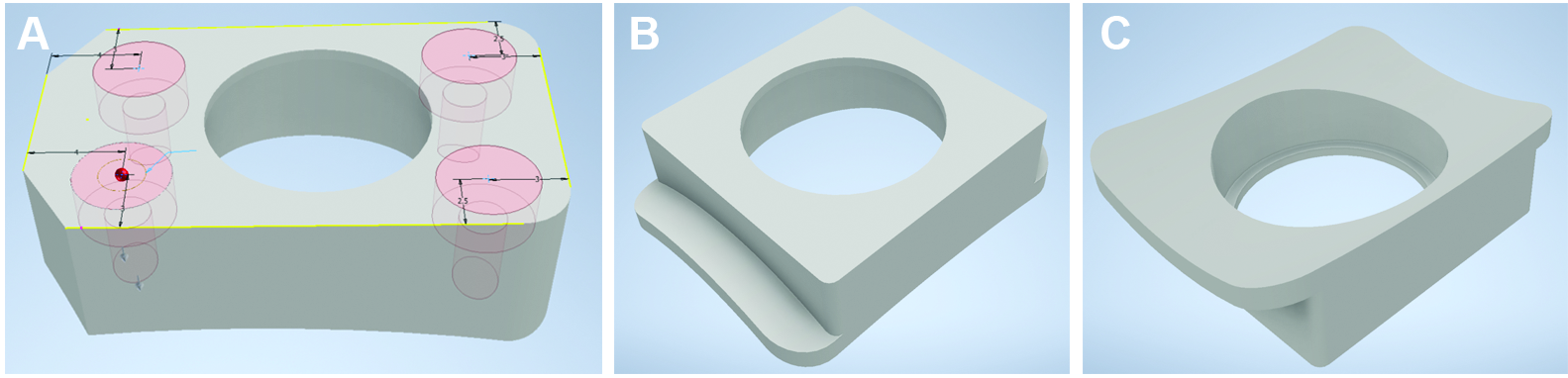
**Supplemental Figure 1: Previous Versions of the Implant.** The first implant was 21.186 mm long to allow for the incorporation of four ports to fit #0-80 screws (A). As our initial version was not easy to secure, we removed the screws and went to a smaller design of 15 mm for the length with 2 mm wings extending from the anterior and posterior edges (B). This second design had a smooth bottom, as compared to the grooves in the bottom of the final design, and it was too long to accommodate a cerebellar anchor screw (C). Both designs were made of MED610.

*Size of the implant (Supplemental Table 1):*

Version 1 of the implant (Supplemental Figure 1A) was large: 21.186 mm along the AP axis, 13.0 mm along the ML axis, and 5.0 mm high. This allowed us to incorporate four anchor screws into the actual 3D printed design. Ultimately, we decided to remove the screws and to shrink the size of the plastic cap because we were not confident that the screws were secure within the skull. Version 2 of the cap remained rectangular but included bowed, 13.000 mm wide x 2.005 mm long x 1.05 mm thick ‘wings’ extending from the ventral aspect of both the anterior and posterior ends, allowing for a greater surface area to anchor the implant with acrylic (Supplemental Figure 1B&C). The new dimensions were 19 mm (15 mm with two 2mm wings) on the AP axis, 13 mm on the ML axis, and 5 mm high. We initially had intended to further secure this implant with an anchor screw over the cerebellum; however the cap was too long. After testing this design on two cohorts of rats (n=14) we observed 5 explants (~33%) within the first month following implant. Ultimately, we reduced the length to 13 mm, plus the two 2.005 mm wings, and added grooves to the ventral surface to increase surface area for the final design (Figure 3). This design allowed ample space for a cerebellar anchor screw, and only one explantation occurred (<5%) with this final version.

**Supplementary Table 1: Comparative Dimensions.** Summary of dimensions for the three main versions of the implant

| **Dimension** | **Version 1 (mm)** | **Version 2 (mm)** | **Final Version (mm)** |
| --- | --- | --- | --- |
| Length of Main Body | 21.186 | 15.0 | 13.0 |
| Width | 13.0 | 13.0 | 13.0 |
| Height at edge | 5.0 | 4.610 | 4.610 |
| Height at midline of front | 5.0 | 2.182 | 2.182 |
| Wing length | - | 2.005 | 2.005 |
| Wing thickness | - | 1.052 | 1.052 |
| Inner cutout diameter | 10.500 | 10.500 | 10.500 |
| Inner lip diameter | 0.600 | 0.600 | 0.600 |
| Inner lip thickness | 0.500 | 0.500 | 0.500 |
| TangoBlackPlus thickness | - | - | 0.500 |
| Groove Spacing | - | - | 0.51 |
| Groove Depth | - | - | 0.175 |

*Material for the Implant:*

Initially, we used an Objet Connex 260v (Stratasys, Eden Prairie, MN) polyjet printer to fabricate the cap from MED610 (Stratasys), an opaque white, biocompatible acrylic. There were no issues with rough edges, and the plastic was sufficiently rigid. However, we realized that our video tracking software could not detect the animal on a light background unless we colored the top of the cap with a water-insoluble permanent marker each day. This required restraining the animals and exposing them to the odor of the marker. Instead, we searched for ways to print the cap using matte black materials. We first tried using a Stratasys F120 series FDM printer to fabricate the cap from black PLA (Stratasys). However, the initial print quality of the caps was not satisfactory. There were rough edges, the cap did not reliably accommodate the metal disc, and the plastic seemed brittle. In our final design, we included a layer of TangoBlackPlus (Stratasys) on top of the white cap. As this material was incompatible with MED610, we printed the main body of the cap from VeroWhitePlus (Stratasys), again using the Objet Connex 260v polyjet printer for fabrication. This final version cut well, was stable, and tracked well in our behavioral experiments.

*Securing the Implant*:

As described above, our initial design incorporated pre-set holes through the plastic, one at each of the four corners of a 21.186 x 13.0 mm plastic cap, to accommodate #0-80 screws for implant stabilization (Supplemental Figure 1A). This initial implant was flat-bottomed and did not account for the size and curvature of an adolescent rat skull. After a small pilot (n=6), it was clear that we could not easily screw down into the skull through the holes. In addition, because they were under the implant, we could not visually verify the quality of the anchor. In our next design, we incorporated the curvature of the skull, reduced the size of the body of the implant to 15.0 x 13.0 mm, and we added 2.005 mm wings on both the anterior and posterior side (Supplemental Figure 1C). These wings were designed to provide extra surface area on the ventral side of the implant for bonding the plastic to the skull. Moreover, as these wings were only 1 mm thick, much thinner than the main implant, they also provided surface area on the dorsal side that could be implanted in the dental acrylic. In addition to the wings, our intention was to incorporate an anchor screw over the cerebellum. However, while the design on the phantom indicated that there was theoretically room, during surgery we found that there was not ample space to place the cerebellar screw. Of the 14 rats implanted with this design, 5 explanted (~33%), indicating that the additional stability of an anchor screw was indeed needed. When we reduced the size of the implant body to 13.0 x 13.0 mm, still incorporating the 2.005 mm wings, we were able to fit a cerebellar skull screw. In addition, the ventral surface of implant was modified to incorporate grooves (Figure 2). Finally, instead of adding dental acrylic as the only compound to create the implant, we first added a thin layer of C&B-Metabond Adhesive Luting Cement (Parkell, Inc., Edgewood, NY). Of 36 caps secured in this manner, only 1 explanted (<5%).

**Additional Information:**

*Applications of the Model to TBI Research*

We designed our model to take advantage of the existing body of literature related to diffuse and mild TBI. Specifically, we chose to permanently implant the metal disc that was developed for the IA model using a custom plastic mold. Then, instead of dropping a weight, we chose to impact the helmet using the electrically driven piston of the CCI device, in order to precisely control impact parameters including the rate of the piston and the depth of penetration into the helmet (Chen et al., 2014; Romine et al., 2014). The advantage of this model is that it offers a high degree of flexibility in the parameters of injury, using devices and equipment that are available to any investigator, and therefore making it a versatile tool for TBI research.

To create a mTBI, we chose to use a 5 mm penetration delivered at 5 m/s. In a traditional open skull CCI, where the piston directly impacts the dura, one would expect to see a significant contusion to both the underlying cortex and hippocampus (reviewed in Siebold, Obenaus, and Goyal 2018). However, unlike the open skull CCI, the implant absorbed much of the force, and the Styrofoam molding allowed for the head to move on impact, ultimately resulting in a mild injury with no detectable hippocampal cell death (Ondek et al., 2020).

One of the limitations of the injury model is that it may be difficult to create a severe TBI. Specifically, as described, the material of the implant absorbs a considerable amount of force as compared to impacting the dura or even the skull directly. In addition, the CCI device used in this study was not designed to strike a metal disc, nor was it designed to move faster than 6 m/s. Therefore, increasing the severity of the mechanical parameters may require an adaptation of the existing to device to both withstand the impact onto a metal disc and incorporate faster rates for the electrically driven piston. As an alternate approach, one could consider the substrate that supports the head during injury. Both the IA model, which uses a highly pliable foam, and the UCLA model, which allows for more free movement, protect the skull from higher forces while also allowing for considerable rotational force (Marmarou et al., 1994; Prins et al., 2010). Perhaps allowing for greater rotation following impact would result in a more significant injury both anatomically and behaviorally. An additional strategy for rotation would be to mount the helmet at an angle. This would allow for greater rotation, in both the coronal and saggital planes. Ultimately, the goal is to transfer sufficient energy from the injury device to the brain to generate the desired injury severity. By using a helmet and implant to diffuse force and a programmable impact device to control injury parameters, it should be possible to modify the existing injury device to generate a sufficient range of forces to generate anything from a mild to a more severe injury, not only in a pediatric or adolescent but also the adult brain.

One of the keys to developing a translational model, particularly of mTBI and repeat TBI, is to keep in mind key biological considerations (reviewed in Bolton-Hall et al., 2019), For example, as the animal ages, the response to an impact of similar magnitude (acceleration and displacement) may vary based on biological factors. For example, as the rat’s age increases, so does skull thickness (Shitaka et al., 2011) with a corresponding decrease in the water content of the brain (Prabhu et al., 2019). In order to initially develop our model, we used specific measurements from the P35 skull. Therefore, it is entirely feasible to either scale up or scale down this model by designing specific implants based on the age of the animal and the curvature of the skull. One of our concerns at the beginning was whether the implant would be stable over time, as the size and shape of the head change with growth. In the current experiment, we found the implants to be stable from P35-P50 when using Metabond as the direct implant to skull connection. However, with a younger animal, it is possible 1) that there would be insufficient skull surface to mount a screw or 2) that, as the skull grew into adulthood, it would weaken the acrylic bond. Either could result in a spontaneous explantation. It is also worth considering whether a smaller width implant with less acrylic might allow for a greater range of force to be applied to the brain, increasing the flexibility of the model for use in both young and older animals.

This model provides the flexibility to alter the inter-injury interval. We chose a 72-hour inter-injury interval with the goal of selecting a clinically relevant time point at which an individual might be considered ready to return to play. In addition, we wanted to ensure that the rats had recovered from any acute physiological effects of injury (e.g. changes in weight and mentation) that may complicate the interpretation of the second insult. Moreover, we were concerned that the repeated injuries within 24 hr could lead to periods of motor or metabolic dysfunction that would result in an animal’s inability to perform on a spatial or object-based learning task. In fact, our pilot data indicated that 100% of animals recovered by 72 hrs, rapidly gaining weight and showing no signs of motor deficit. In fact, the animals were visibly no different from sham controls within 30 min following injury. Therefore, it is possible to considerably shorten the inter-injury interval to days, if not hours, without risking the loss of animals due to severity of injury or significant motor deficits.

*Conclusions*

Over the last decade, there has clearly been a push to develop more clinically relevant models of mTBI and repeat mTBI. We observed that many of the described sources of repeat mTBI were sports- or military-related activities, in which individuals often wear helmets to diffuse the force of an impact. With these injuries in mind, we incorporated the metal disc used in the IA model to create a more diffuse injury with the programmable and electronically controlled CCI device to deliver a reliable and rapid insult directly to an implanted helmet. In order to account for differences in skull shape and size through development, we used a rat skull to create a customized implant that could be mounted to the skull days prior to injury and would last throughout the duration of the experiment without any further surgical procedures or significant anesthesia. Our goal was to make a highly adaptable implant, allowing for injury intervals ranging from minutes to weeks, and for the investigator to evaluate the effect of any number of repeat TBI without having to perform additional survival surgery. Future studies will need to critically evaluate the material that initially supports the head but also allows for axial rotation following injury, to use a device that allows for a greater range of forces to be applied to the implant, and to carefully consider appropriate control groups, including evaluating sex as a potential biological variable. Ultimately, more translatable models, particularly ones that can be used similarly on developing and adult rats, are necessary to advance our understanding of mechanisms related to of mTBI and repeat TBI and to identify potential targets for intervention in those patients that develop lasting sequela in the months-to-years following injury.

**References:**

Bolton-Hall, A.N., Hubbard, W.B., and Saatman, K.E. (2019). Experimental Designs for Repeated Mild Traumatic Brain Injury: Challenges and Considerations. J. Neurotrauma *36*, 1203–1221.

Chen, J.-Q., Zhang, C.-C., Lu, H., and Wang, W. (2014). Assessment of traumatic brain injury degree in animal model. Asian Pac J Trop Med *7*, 991–995.

Greco, T., Hovda, D., and Prins, M. (2013). The effects of repeat traumatic brain injury on the pituitary in adolescent rats. J. Neurotrauma *30*, 1983–1990.

Heath, D.L., and Vink, R. (1995). Impact acceleration-induced severe diffuse axonal injury in rats: characterization of phosphate metabolism and neurologic outcome. J. Neurotrauma *12*, 1027–1034.

Huh, J.W., and Raghupathi, R. (2007). Chronic cognitive deficits and long-term histopathological alterations following contusive brain injury in the immature rat. J. Neurotrauma *24*, 1460–1474.

Huh, J.W., Franklin, M.A., Widing, A.G., and Raghupathi, R. (2006). Regionally distinct patterns of calpain activation and traumatic axonal injury following contusive brain injury in immature rats. Dev. Neurosci. *28*, 466–476.

Marmarou, A., Foda, M.A., van den Brink, W., Campbell, J., Kita, H., and Demetriadou, K. (1994). A new model of diffuse brain injury in rats. Part I: Pathophysiology and biomechanics. J. Neurosurg. *80*, 291–300.

Ondek, K., Brevnova, O., Jimenez-Ornelas, C., Vergara, A., Zwienenberg, M., and Gurkoff, G. (2020). A new model of repeat mTBI in adolescent rats. Exp. Neurol. 113360.

Prabhu, R.K., Begonia, M.T., Whittington, W.R., Murphy, M.A., Mao, Y., Liao, J., Williams, L.N., Horstemeyer, M.F., and Sheng, J. (2019). Compressive Mechanical Properties of Porcine Brain: Experimentation and Modeling of the Tissue Hydration Effects. Bioengineering (Basel) *6*.

Prins, M.L., and Giza, C.C. (2012). Repeat traumatic brain injury in the developing brain. Int. J. Dev. Neurosci. *30*, 185–190.

Prins, M.L., Hales, A., Reger, M., Giza, C.C., and Hovda, D.A. (2010). Repeat traumatic brain injury in the juvenile rat is associated with increased axonal injury and cognitive impairments. Dev. Neurosci. *32*, 510–518.

Romine, J., Gao, X., and Chen, J. (2014). Controlled cortical impact model for traumatic brain injury. J Vis Exp e51781.

Shitaka, Y., Tran, H.T., Bennett, R.E., Sanchez, L., Levy, M.A., Dikranian, K., and Brody, D.L. (2011). Repetitive closed-skull traumatic brain injury in mice causes persistent multifocal axonal injury and microglial reactivity. J. Neuropathol. Exp. Neurol. *70*, 551–567.

Siebold, L., Obenaus, A., and Goyal, R. (2018). Criteria to define mild, moderate, and severe traumatic brain injury in the mouse controlled cortical impact model. Exp. Neurol. *310*, 48–57.

Yoshiyama, Y., Uryu, K., Higuchi, M., Longhi, L., Hoover, R., Fujimoto, S., McIntosh, T., Lee, V.M.-Y., and Trojanowski, J.Q. (2005). Enhanced neurofibrillary tangle formation, cerebral atrophy, and cognitive deficits induced by repetitive mild brain injury in a transgenic tauopathy mouse model. J. Neurotrauma *22*, 1134–1141.
